# Dietary Supplementation with Resveratrol Attenuates Serum Melatonin Level, Pro-Inflammatory Response and Metabolic Disorder in Rats Fed High-Fructose High-Lipid Diet under Round-the-Clock Lighting

**DOI:** 10.3390/pathophysiology30010005

**Published:** 2023-02-19

**Authors:** Yurii Frenkel, Valerii Cherno, Heorhii Kostenko, Hitesh Chopra, Rupesh K. Gautam, Vitalii Kostenko

**Affiliations:** 1 Department of Anatomy, Clinical Anatomy, Operative Surgery, Pathomorphology and Forensic Medicine, Petro Mohyla Black Sea National University, 54000 Mykolayiv, Ukraine; 2 Department of Pathophysiology, Poltava State Medical University, 36011 Poltava, Ukraine; 3 Chitkara College of Pharmacy, Chitkara University, Punjab 140401, India; 4 Department of Pharmacology, Indore Institute of Pharmacy, Rau, Indore 453331, India

**Keywords:** resveratrol, high-fructose high-lipid diet, light-dark cycle, melatonin, pro-inflammatory activity, oxidative stress, metabolic disorder, rats

## Abstract

This study aims to investigate the effect of resveratrol on systemic inflammatory response and metabolic disorder in rats fed a high-fructose high-lipid diet (HFHLD) and exposed to round-the-clock lighting (RCL). 21 adult male Wistar rats were randomly divided into 3 groups: control (group 1, *n* = 7); HFHLD for 8 weeks + round-the-clock lighting (RCL) (group 2, *n* = 7); HFHLD + RCL + Resveratrol (in a daily dose of 5 mg/kg intragastrically (group 3, *n* = 7). Results show that the combined effect of HFHLD and RCL reduces the serum melatonin (*p* < 0.001) and accelerates pro-inflammatory activities, oxidative stress, and metabolic disorder. There is a significant increase in the serum tumour necrosis factor-alpha (TNF-α) and C-reactive protein (CRP) (both *p* < 0.001), blood malondialdehyde—thiobarbituric acid adducts (MDA-TBA_2_) (*p* < 0.001), serum glucose *(p* < 0.01), insulin concentration, and the homeostatic model assessment insulin resistance (HOMA-IR) index (both *p* < 0.001), serum with very low-density lipoprotein (VLDL), and triacylglycerol (TAG) (both *p* < 0.001). At the same time, the decrease in the serum high-density lipoprotein (HDL) level (*p* < 0.001) is observed in the HFHLD + RCL group compared to the control. In the HFHLD + RCL + Resveratrol group, hypomelatonaemia (*p* < 0.001), pro-inflammatory actions, oxidative stress, and metabolic disorder were mitigated. Resveratrol can cause a significant rise in the serum melatonin and reduce serum TNF-α and CRP levels (both *p* < 0.001), blood MDA-TBA_2_ (*p* < 0.001), serum glucose (both *p* < 0.01), insulin concentration, and HOMA-IR (both *p* < 0.001), serum VLDL and TAG (both *p* < 0.001) compared to the group 2, while serum HDL level increases (*p* < 0.01). Resveratrol attenuates pro-inflammatory responses and prevents considerable metabolic disorder in rats fed HFHLD under RCL.

## 1. Introduction

Substantial changes in the daily light-dark cycle can result in the disorganisation of the circadian system, including melatonin rhythm alterations. The relevance of this issue is conditioned by the changes reshaping the sleep/work cycle in industrialised countries and the growing tendency to a night work regime with the rise in the exploitation of visual display units, smartphones, and light-emitting diode lighting that cause a shift in the light spectrum toward artificial lighting sources [1].

In the long term, light-dependent disorganisation of the circadian system appears to be very detrimental to health. A number of epidemiological studies have shown that under these conditions there is a significant increase in rates of several diseases, including metabolic syndrome, type 2 diabetes, obesity, cardiovascular diseases, mood disorders, cancer, and age-related risks [2,3,4]. Voluminous experimental and clinical studies evidence the role of hypomelatonaemia in the mechanisms contributing to the development of carbohydrate and lipid metabolic disorders, systemic inflammation, endothelial dysfunction, and nitro-oxidative stress [5].

In our opinion, these disorders can vary considerably under the combined impact of light-dependent disorganisation of the circadian system and diet. Recent reports demonstrate the impact of diets on serum melatonin and its circadian fluctuation. For instance, the capability of certain concentrations of caffeine and alcohol, as well as deficiency of some nutrients (folate, magnesium, and zinc), to reduce the nocturnal production of melatonin, but this effect is minor compared with the light–dark cycle [6].

Our more recent studies on rats have proven that the melatonin concentration in the blood serum can considerably decrease under combined exposure to round-the-clock lighting (RCL) and a high-fructose high-lipid diet (HFHLD) for 60 days [7]. The administration of melatonin under this condition partly lessens the manifestation of carbohydrate and lipid metabolism disorders and signs of nitro-oxidative stress in the skeletal muscles and the liver of rats, though a homeostatic model assessment insulin resistance (HOMA-IR) index under this condition demonstrates no significant changes [8]. It is evident that under these conditions the normalisation of the melatonin level is not sufficient to correct metabolic disorder, which can be achieved by suppressing the nuclear factor kappa-light-chain-enhancer of activated B cells (NF-κB) or by inducing the nuclear-factor-E2-related factor-2 (Nrf2), antagonistic to NF-κB [9,10]. The best safety profile among agents that can inhibit NF-κB and/or activate Nrf2 is found in plant polyphenols, particularly in bioflavonoids, epigallocatechin-3-gallate [11,12], and quercetin [13,14].

Resveratrol (3,4′,5-trihydroxy-trans-stilbene), a natural phytoalexin, is also able to concurrently suppress NF-κB and activate Nrf2–antioxidant response element (ARE) signalling pathway [15,16], alleviate NF-κB-dependent pro-inflammatory hypercytokinaemia and endothelium dysfunction (in higher degree compared to quercetin) [17], and, in combination with melatonin, enhances antioxidant activity and decreases gene expression of the insulin-regulated glucose transporter GLUT4, sirtuin 1 (SIRT1) and peroxisome proliferator-activated receptor gamma coactivator 1-alpha (PGC-1α) in heart tissue in a diabetic aged rat model [18].

Given that the role of transcriptional factors NF-κB and Nrf2 is associated with modifying the mammalian circadian clock [19,20], it seems important to focus on the capability of resveratrol to modulate serum melatonin levels under the co-effect of RCL and HFHLD. Another reason to carry out this study is to investigate the effect produced by resveratrol on the systemic inflammatory response and carbohydrate and lipid metabolism under the combined action of the circadian system disorganisation and “western diet”, considering the fact that efficacy of specific NF-κB inhibitor ammonium pyrrolidine diothiocarbamate or Nrf2 inducer dimethyl fumarate is limited because of their toxicity [9,10].

Therefore, this study aims to investigate the effect of resveratrol on serum melatonin levels, systemic inflammatory response, and metabolic disorder in rats fed a HFHLD and exposed to RCL.

## 2. Materials and Methods

### 2.1. Chemicals

Crystalline D-Fructose (≥99.5%, Ph. Eur.) was purchased from ADM, Turkey, while resveratrol (3,4′,5-Trihydroxy-trans-stilbene,5-[(1E)-2-(4-Hydroxyphenyl)ethenyl]-1,3- benzenediol, ≥99%)was obtained from Merck Life Science, Poland. Diagnostic kits for determining insulin, tumour necrosis factor-alpha (TNF-α), and C-reactive protein (CRP) in the blood serum were purchased from MyBioSource.com, USA. Analytical kits for measuring glucose and assessing the lipid profile in the blood serum were procured from Filisit-Diagnostics, Ukraine.

### 2.2. Experimental Subjects

The 21 adult male Wistar rats (weight: 235 ± 20 g) used for this study were bred in an experimental biological clinic (vivarium) at the Petro Mohyla Black Sea National University, Mykolayiv, Ukraine. The animals were kept under standard environmental conditions (air temperature: +22 ± 2 °C, air humidity: 30–60%). The animals had free access to water and rodent pellets. Prior to the commencement of the study, bioethical approval was obtained from the Commission on Bioethics of Petro Mohyla Black Sea National University, Mykolayiv, Ukraine.

### 2.3. Experimental Design

The rats were randomly divided into 3 groups: control (group 1, *n* = 7); High-Fructose High-Lipid Diet (HFHLD) + round-the-clock lighting (RCL) (group 2, *n* = 7); HFHLD + RCL + Resveratrol (group 3, *n* = 7). Group 1 was fed standard chow (Table 1) and kept on a 12/12 h light/dark cycle. Group 2 waskept on a HFHLD for 8 weeks and exposed to RCL. Group 3 received resveratrol in a daily dose of 5 mg/kg [21] intragastrically. Resveratrol was administered together with carbohydrates (20% aqueous solution of fructose) whichincreased the solubility and bioavailability of stilbenoids [22]. Rats fromthe first two groups, instead of receiving resveratrol, were given 1 mL of a 20% solution of fructose intragastrically as a “placebo”.

The rats were kept on a HFHLD for 2 months: the animals received a 20% aqueous solution of fructose for drinking and ahigh-lipid diet, whose components and content are given in Table 1. Fromthe 30th day of the experiment, the rats were exposed to RCL with an intensity of 1500 lx over the next 30 days, as previously reported [23].

After the experiment, the rats were sacrificed under thiopental anaesthesia in the morning (8.00–10.00), minimizing the effect of daily fluctuations in pineal melatonin secretion. The animals were given thiopental sodium (50 mg/kg, intraperitoneally, manufacturer: Kyivmedpreparat, Arterium Corporation, Kyiv, Ukraine). After that, they were dissected, and blood was taken via cardiac puncture sample bottles containing lithium heparin (30 IU per 1 mL of blood) (article LG3902, obtained from Sky Medica, Kyiv, Ukraine). Then heparinised blood was centrifuged (3000× *g*, 15 min) at room temperature. Each sample’s separated top layer of serum was used for the analysis. 

### 2.4. Biochemical and Enzyme-Linked Immunosorbent Assays

To assess the serum melatonin levels, TNF-α, CRP, and insulin we used highly sensitive and specific ELISA kits for rat samples. Optical density readings were taken with awavelength of 450 nm (Stat Fax 2100 Microplate Reader, Awareness Technology, Inc., Palm City, FL, USA).

To measure secondary products of lipid peroxidation (LPO) in the blood—malondialdehyde (MDA)—thiobarbituric acid (TBA) adducts (MDA-TBA_2_) with maximum light absorption of 532 nm, we used a spectrophotometer ULAB 101 (China). A rise in their concentration after 1.5 h incubation in a pro-oxidant ascorbate-iron buffer (pH = 7.4; 1 litre of the buffer contained 1.9 g tris-(2-hydroxy-methyl)-aminomethane hydrochloride, 50 mL 0.1 N HCl, 1.4 g ascorbic acid, and 32 mg FeSO_4_ × 7H_2_O) and was used to measure the general antioxidant blood potential (the ability of the blood to resist LPO under the aggressive pro-oxidant environment) [24].

Serum glucose, total cholesterol (CH), high-density lipoprotein (HDL), low-density lipoprotein (LDL), very low-density lipoprotein (VLDL), and triacylglycerol (TAG) concentrations were measured using enzymatic methods employing photometric equipment for measuring the optical density of materials that can measure the optical density of solutions at a wavelength of 490–600 nm (spectrophotometer ULAB 101, China) and standard laboratory reagent kits.

Insulin resistance was assessed by the homeostatic model assessment insulin resistance (HOMA-IR) index using the equation: HOMA-IR = fasting glucose (mmol/L) × fasting insulin (μU/mL)/22.5 [25].

### 2.5. Statistical Analysis

The findings were statistically analysed using the Microsoft Office Excel software package with the Real Statistics 2019 extension. We exploited the Shapiro-Wilk test to verify the normality of variances. The arithmetic mean and standard error of mean (SEM) were computed. The results are presented as mean ± SEM. Assuming that all samples had a normal distribution, we used the parametric analysis of variance (ANOVA), which was followed by a pairwise comparison of groups using the Student’s t-test for independent samples and Tukey’s honestly significant difference analysis. Multiple comparisons were avoided by employing the Dunn—Šidák correction. *p* < 0.05 was used to determine whether the differences between the arithmetic means were significant.

## 3. Results

### 3.1. Effects of Resveratrol on the Melatonin Level in the Serum of the Rats Fed a High-Fructose High-Lipid Diet under Round-the-Clock Lighting

**Figure 1:**
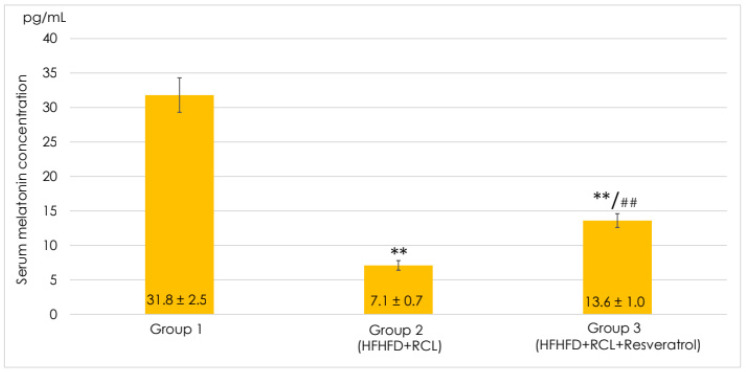
Serum Melatonin, *n* = 7. Values were expressed as Mean ± SEM. Control, HFHLD (high-fructose high-lipid diet) + RCL (round-the-clock lighting), and HFHLD + RCL + Resveratrol; ** *p* < 0.001 is significant compared to group 1; ## *p* < 0.001 is significant compared to group 2.

Under the combined effect of RCL and HFHLD, the serum melatonin concentration significantly decreased by 4.5 times, achieving 7.1 ± 0.7 pg/mL (*p* < 0.001) (Figure 1). The dietary supplementation with resveratrol under RCL and HFHLD resulted in the growth of the melatonin level, which was 1.9 times higher than the respective values in group 2 (*p* < 0.001).

### 3.2. Effects of Resveratrol on the Systemic Inflammatory Response Indices in the Serum of Rats Fed a High-Fructose High-Lipid Diet under Round-the-Clock Lighting

The animals exposed to RCL and kept on HFHLD demonstrated considerable deterioration of systemic inflammatory response indices, such as serum concentrations of the TNF-α, a pro-inflammatory cytokine, CRP, and an acute phase reactant (Table 2). 

The serum TNF-α and CRP levels in rats with RCL and HFHLD were 3.2-fold and 3.1-fold higher, respectively, than in the control animals (both *p* < 0.001).

The administration of resveratrol during the RCL and HFHLD combined exposure led to a statistically significant reduction in the TNF-α and CRP concentration by 2.5 and 2.4 times, respectively, compared to the results in group 2 (both *p* < 0.001).

### 3.3. Effects of Resveratrol on Lipid Peroxidation in the Blood of Rats Fed a High-Fructose High-Lipid Diet under Round-the-Clock Lighting

Simultaneous action of the RCL and HFHLD was accompanied by the changes in LPO and general antioxidant blood potential in the rats’ blood (Table 3). When compared to the corresponding values in the control group, the MDA-TBA_2_ concentration under RCL and HFHLD nearly doubled: it grew 2.1-fold before the incubation and 1.9-fold after the incubation (both *p* < 0.001). The MDA-TBA_2_ increment throughout incubation in the pro-oxidant buffer solution was 1.8 times higher than the results in the control rats (*p* < 0.01), indicating a significant decline in general antioxidant blood potential.

Resveratrol administered under RCL and HFHLD lowered the MDA-TBA_2_ level in the blood by 1.8 times (before incubation) and by 1.7 times (after incubation) in comparison to the group 2 values (both *p* < 0.001). The MDA-TBA_2_ increment over incubation in a pro-oxidant buffer solution was 1.7 times less than the respective values in group 2 (*p* < 0.01), which evidences the growth in general antioxidant blood potential under the dietary supplementation of resveratrol.

### 3.4. Effects of Resveratrol on Carbohydrate Metabolism in the Serum of Rats Fed a High-Fructose High-Lipid Diet under Round-the-Clock Lighting

The animals exposed to RCL and kept on HFHLD demonstrated considerable deterioration of carbohydrate metabolism (Table 4). There was a 1.4-fold difference between the serum glucose levels and the corresponding values in group 1 (*p* < 0.01). The serum insulin concentration and HOMA-IR were 3.7 times higher compared to the control group (both *p* < 0.001).

Resveratrol administered under RCL and HFHLD combination reduced the glucose level, which was 1.5 times inferior to the result in group 2 (*p* < 0.01). In comparison to the results in group 2, the serum insulin level and HOMA-IR fell 2.6 and 2.8 times, respectively (both *p* < 0.001).

### 3.5. Effects of Resveratrol on Lipid Profile in the Serum of Rats Fed a High-Fructose High-Lipid Diet under Round-the-Clock Lighting

The animals, when exposed to RCL and receiving HFHLD, demonstrated a considerable 2.7-fold fall in serum HDL compared to the respective value in the control rats (*p* < 0.001) (Table 5). On the other hand, VLDL and TAG levels considerably rose and were 3.3 and 3.2 times higher than in group 1, respectively (both *p* < 0.001).

The HDL concentration was increased by dietary resveratrol supplementation under RCL and HFHLD combination and was 2.1 times higher than the results in group 2 (*p* < 0.01). When compared to the results in group 2, the levels of VLDL and TAG decreasedby1.9 times for each (both *p* < 0.001).

## 4. Discussion

Earlier, we demonstrated that the development of hypomelatoninaemia naturally occurs in rats due to long-term RCL exposure and does not manifest when the rats are kept on HFHLD. However, when these factors act together, the melatonin level is significantly reduced compared to the effects resulting from the separate use of the above-mentioned factors [7]. This confirms the ability of alimentary factors under certain conditions to influence melatonin secretion, which was discovered by other researchers [3]. Dietary components such as glucose, sodium, ethanol, and caffeine have been shown to affect circadian rhythms and melatonin production by altering the expression of circadian oscillator proteins [3]. We can presume that other carbohydrates (besides glucose) and lipids are also capable of reducing pineal melatonin production through similar mechanisms.

It is noteworthy that the dietary supplementation with resveratrol over RCL and HFHLD significantly elevated the serum melatonin level compared with the findings of group 2. Earlier studies on the diabetic-aged rat model demonstrated that the effect of resveratrol and melatonin administered separately and their combination could increase SIRT1 gene expression in heart tissue [18] that can lead to the reduction of the activity of several pro-inflammatory and pro-oxidant transcription factors, such as NF-κB, STAT3, FOXO, and p53 [26]. The NF-κB suppression restricts the transcription of the crucial enzyme in pineal melatonin synthesis—arylalkylamine N-acetyltransferase [27]. This points out that resveratrol can be considered as both an inducer of melatonin synthesis and a compound that has a synergic impact along with this hormone on the immune system and metabolism due to their effects on the same transcription factors.

Some studies have revealed the association between systemic inflammatory response and diets rich in fructose [28] and fats [29]. For instance, fructose intake increases the pro-inflammatory cytokines, intestinal permeability, and lipid accumulation in the liver, and increases pro-inflammatory cytokines [28]. Elevated free fatty acids and cytokines can activate the inhibitory kappa B kinase (IKK) complex and NF-κB both through activating Toll-like receptors and through inducing cellular stresses (oxidative or/and endoplasmic reticulum) [29]. High fructose consumption can activate NF-κB via sphingosine kinase 1/sphingosine-1-phosphate induction [30]. Excessive lighting in the dark phase leads to the perturbation of clock genes (CLOCK, PER1, PER2) in humans and rodents by increasing immune activation and producing pro-inflammatory cytokines, even in the absence of immune challenge [31,32]. Some proteins of the circadian oscillator, and, in particular, CLOCK, are known to be involved in the NF-κB activation [33].

In fact, according to our findings, the rats exposed to RCL and kept on HFHLD demonstrated an increase in TNF-α and CRP, which are markers of the systemic inflammatory response. The obtained results on the statistically significant reduction in the serum TNF-α and CRP under RCL and HFHLD under the resveratrol administration are supported by the data from randomised controlled trials according to which dietary supplementation with resveratrol dramatically decreased TNF-α and CRP levels [34]. Resveratrol is supported as an adjuvant to pharmacologic therapy of metabolic disorders, significantly improving inflammatory markers.

Previously we have found out that the simultaneous effect of RCL and HFHLD leads to more marked metabolic disorders, such as LPO, and a decrease in the general antioxidant blood potential in the blood of rats, hyperinsulinaemia, hypo-α-lipoproteinaemia, hypertrialcylglycerolaemia, and increased visceral fat mass, in comparison with the animals kept on the HFHLD only [7]. This study demonstrated that resveratrol administered under RCL and HFHLD reduces the MDA-TBA_2_ level and its increment over 1.5-h incubation in pro-oxidant ascorbate-iron buffer that points out the LPO restrain and growth in general antioxidant blood potential. It has been suggested that the strong antioxidant properties of resveratrol allow it to reduce oxidative stress and inhibit free radicals, especially those produced by LPO. The findings of this study are consistent with other published results, which demonstrated that rats fed with various levels of resveratrol had decreased serum LPO products. For instance, 4-month supplementation of 0.04% and 0.06% resveratrol significantly lowered MDA concentration in the serum of rats fed high fructose diet (63%) [35]. A 10-week resveratrol diet supplementation (at a daily dose of 30 mg/kg·bw) significantly decreased hepatic MDA, improved its superoxide dismutase and catalase, and reduced glutathione values in rats fed HFHLD; additionally, serum total antioxidant capacity was insignificantly affected compared to the HFHLD group [36]. Resveratrol is known as an antioxidant exerting a dual effect: it can increase the activity of antioxidant enzymes (manganese-containing superoxide dismutase, catalase, glutathione peroxidase, glutathione S-transferase, and glutathione reductase) and can act as a scavenger of superoxide, hydroxyl and other free radicals that prevents LPO in cell membranes and DNA lesions [37]. It is known that the expression of the genes encoding the aforementioned antioxidant enzymes is associated with the activation of the Nrf2–ARE by resveratrol [38].

Under RCL, the antioxidant effect of resveratrol can also be associated with its capability, discovered in this study, to increase serum melatonin levels. This hormone, known for its direct antiradical properties [39], promotes the expression of a number of antioxidant enzymes (superoxide dismutase, glutathione peroxidase) [40].

The dietary supplementation with resveratrol under RCL and HFHLD produces sufficient impact on carbohydrate and lipid metabolism under the experimental conditions by lowering serum glucose and insulin, HOMA-IR, as well as serum VLDL and TAG, and increasing HDL level compared with the findings in group 2.

Our results correspond with data on the effect of resveratrol on carbohydrate and lipid metabolism, demonstrated under long-term resveratrol administration. Thus, resveratrol considerably improves the lipid profile (significantly declines the VLDL and TAGs concentrations, normalizes CH and LDL levels, and increases the HDL level), insulin sensitivity, and hepatic mRNA expression of peroxisome proliferator-activated receptor alpha (PPARα), and diminishes hepatic NF-κB and morphological characteristics of liver steatosis [36]. The role of disorganisation of the circadian system in the mechanisms of metabolic disorders is confirmed by the data on the capability of resveratrol to abolish 11-week high-fat diet-induced circadian desynchrony and ameliorate the impaired lipid profiles, the plasma leptin rhythmities in mice that, as we suggest, can be associated with its impact on the expression of clock genes (BMAL1, CLOCK, and PER2) and clock-controlled lipid metabolism-related genes (SIRT1, PPARα, SREBP-1c, ACC1, and FAS) [41].

Obviously, the mitigation of pro-inflammatory responses and metabolic disorder under the combined pathogenic impact of a high-calorie carbohydrate-lipid diet and light-dependent disruption of the biological clocks results from the effect caused by resveratrol on key signalling pathways involved in the regulation of immune response, inflammation, oxidative stress, carbohydrate and lipid metabolism, and circadian oscillator. This multidirectional action of resveratrol favourable distinguishes it from other polyphenols.

We are aware of some limitations in this study. The rat model is not a complete simulation of the pathological processes in patients who experience the pathogenic effects of the western lifestyle, particularly a high-calorie carbohydrate-lipid diet, and light-dependent disruption of the biological clocks. Therefore, additional clinical data are required to support the results so far. Since this study investigated the effects of resveratrol under the combined action of two factors (HFHLD and RCL), which can cause more pronounced inflammatory and metabolic disorders, as shown in our previous paper [7], the assessment of effects produced by resveratrol under separate action of these factors requires special experimental design, including additional control groups. Moreover, the measurement of serum melatonin in the morning does not reveal the regularities of its circadian rhythmicity in rats during the experiment. Determining the MDA-TBA_2_ increment over the blood incubation in pro-oxidant buffer solution evaluated only the general antioxidant blood potential regardless of the contribution from enzymatic and low-molecular antioxidant compounds that requires further in-depth investigation.

## 5. Conclusions

The administration of resveratrol considerably restrains the fall of serum melatonin, reduces serum TNF-α and CRP levels, blood MDA-TBA_2_, serum glucose and insulin concentrations, HOMA-IR, serum VLDL, and TAG levels, increases serum HDL, and thereby prevents pro-inflammatory activities, LPO and metabolic disorder, as well as improving general antioxidant blood potential in rats fed a high-fructose high-lipid diet under round-the-clock lighting.

## Figures and Tables

**Table 1:** Components and contents in standard and high-lipid diet.

Standard Rat Chow		High-Lipid Diet	
Nutrients	g/kg Total	Nutrients	g/kg Total
Protein	160	Protein	94
Fat	70	Fat	313
Carbohydrate	480	Carbohydrate	466
Fibre	68	Fibre	14
Sodium	2.7	Sodium	4
Vitaminand mineral mix	36	Vitaminand mineral mix	40
		Ingredients:	
		Refined wheat flour	450
		Skimmed milk powder	200
		Table margarine 82% fat	200
		Starch	100
		Peroxidised sunflower oil	40
		Sodium chloride	10
Total calorie	2720 kcal/kg	Total calorie	4477 kcal/kg

**Table 2:** Effects of resveratrol on the systemic inflammatory response indices in the serum of rats fed a high-fructose high-lipid diet (HFHLD) under round-the-clock lighting (RCL).

Groups/Parameters	TNF-α (pg/mL)	CRP (ng/mL)
1. Group 1 (Control), *n* = 7	34.0 ± 2.0	4.1 ± 0.1
2. Group 2 (HFHLD + RCL), *n* = 7	109.8 ± 6.0 **	12.8 ± 0.3 **
3. Group 3 (HFHLD + RCL + Resveratrol), *n* = 7	43.6 ± 4.9 ##	5.3 ± 0.3 *,##

Note: The table represents the mean ± SEM; * *p* < 0.01, and ** *p* < 0.001 is significant compared to group 1; ## *p* < 0.001 is significant compared to group 2; TNF-α—Tumour necrosis factor-alpha; CRP—C-reactive protein.

**Table 3:** Effects of resveratrol on lipid peroxidation (LPO) in the blood of rats fed a high-fructose high-lipid diet (HFHLD) under round-the-clock lighting (RCL).

Groups/Parameters	MDA-TBA_2_ (µmol/L)		
	Before Incubation	After Incubation	Increment over Incubation Time
1. Group 1 (Control), *n* = 7	11.2 ± 0.9	25.4 ± 2.0	14.2 ± 2.4
2. Group 2 (HFHLD + RCL), *n* = 7	23.0 ± 0.6 **	48.3 ± 2.1 **	25.3 ± 1.9 *
3. Group 3 (HFHLD + RCL + Resveratrol), *n* = 7	12.6 ± 0.9 ##	27.7 ± 1.9 ##	15.1 ± 2.2 #

Note: The table represents the mean ± SEM; * *p* < 0.01, and ** *p* < 0.001 is significant compared to group 1; # *p* < 0.01, and ## *p* < 0.001 is significant compared to group 2; MDA-TBA_2_—Malondialdehyde—thiobarbituric acid adducts.

**Table 4:** Effects of resveratrol on carbohydrate metabolism in the serum of rats fed a high-fructose high-lipid diet (HFHLD) under round-the-clock lighting (RCL).

Groups/Parameters	Glucose (mmol/L)	Insulin (μU/mL)	HOMA-IR
1. Group 1 (Control), *n* = 7	4.94 ± 0.24	1.5 ± 0.2	0.32 ± 0.06
2. Group 2 (HFHLD + RCL), *n* = 7	6.89 ± 0.25 *	5.5 ± 0.2 **	1.17 ± 0.04 **
3. Group 3 (HFHLD + RCL + Resveratrol), *n* = 7	4.45 ± 0.18 #	2.1 ± 0.1 ##	0.42 ± 0.03 ##

Note: The table represents the mean ± SEM; * *p* < 0.01, and ** *p* < 0.001 is significant compared to group 1; # *p* < 0.01, and ## *p* < 0.001 is significant compared to group 2; HOMA-IR—Homeostasis Model Assessment of Insulin Resistance.

**Table 5:** Effects of resveratrol on lipid profile in the serum of rats fed a high-fructose high-lipid diet (HFHLD) under round-the-clock lighting (RCL).

Groups/Parameters	Total CH (mmol/L)	HDL (mmol/L)	LDL (mmol/L)	VLDL (mmol/L)	TAG (mmol/L)
1. Group 1 (Control), *n* = 7	2.39 ± 0.29	0.63 ± 0.04	1.47 ± 0.29	0.29 ± 0.02	0.65 ± 0.05
2. Group 2 (HFHLD + RCL), *n* = 7	2.62 ± 0.30	0.23 ± 0.02 **	1.43 ± 0.29	0.96 ± 0.04 **	2.10 ± 0.10 **
3. Group 3 (HFHLD + RCL + Resveratrol), *n* = 7	2.28 ± 0.22	0.48 ± 0.02 *,#	1.29 ± 0.22	0.51 ± 0.03 **,##	1.11 ± 0.06 **,##

Note: The table represents the mean ± SEM; * *p* < 0.01, and ** *p* < 0.001 is significant compared to group 1; # *p* < 0.01, and ## *p* < 0.001 is significant compared to group 2; CH—Cholesterol; HDL—High-density lipoprotein; LDL—Low-density lipoprotein; VLDL—Very low-density lipoprotein; TAGs—Triacylglycerol.

## Data Availability

The datasets used and/or analysed during the current study are available from the corresponding author upon reasonable request.
